# Enzyme-linked immunosorbent assay of epidermal growth factor receptor in lung cancer: comparisons with immunohistochemistry, clinicopathological features and prognosis.

**DOI:** 10.1038/bjc.1998.448

**Published:** 1998-07

**Authors:** P. Pfeiffer, E. Nexø, S. M. Bentzen, P. P. Clausen, K. Andersen, C. Rose

**Affiliations:** Department of Oncology, Odense University Hospital, Denmark.

## Abstract

The prognostic role of epidermal growth factor receptor (EGFR) remains controversial in patients with lung cancer. Previous assays for EGFR have primarily been qualitative or, at best, semiquantitative. In the present study, using fresh-frozen tissue from 190 unselected lung cancer patients, quantification of EGFR (EGFR(ELISA)) using a recently developed enzyme-linked immunosorbent assay (ELISA) technique was compared with results (EGFR(IHC)) obtained using immunohistochemistry (IHC). Correlation between results obtained by the two different techniques was highly significant (r(s) = 0.63, P < 0.001, n = 190). This correlation improved even further (r(s) = 0.76) when sections were estimated using an IHC score that took into account percentage staining, intensity and relative tumour area. Furthermore, the relationship between clinicopathological features and prognosis was identical for the two methods. The expression of EGFR was highest in squamous cell carcinomas, but it was not correlated with other characteristics such as age, sex, histological grading, stage or prognosis. We conclude that evaluation of EGFR content using IHC and ELISA produces comparable results.


British Journal of Cancer (1998) 78(1), 96–99
© 1998 Cancer Research Campaign

### Enzyme-linked immunosorbent assay of epidermal growth factor receptor in lung cancer: comparisons with immunohistochemistry, clinicopathological features and prognosis

**P Pfeiffer[1], E Nexø[2], SM Bentzen[3], PP Clausen[4], K Andersen[5] and C Rose[1]**

[1]Department of Oncology[1], Odense University Hospital, Denmark; [2]Department of Clinical Biochemistry KH and the Danish Cancer Society; [3]Department of Experimental Clinical Oncology, Aarhus University Hospital, Denmark; Departments of [4]Pathology and [5]Thoracic Surgery, Odense University Hospital, Denmark

**Summary** The prognostic role of epidermal growth factor receptor (EGFR) remains controversial in patients with lung cancer. Previous assays for EGFR have primarily been qualitative or, at best, semiquantitative. In the present study, using fresh-frozen tissue from 190 unselected lung cancer patients, quantification of EGFR ($EGFR_{ELISA}$) using a recently developed enzyme-linked immunosorbent assay (ELISA) technique was compared with results ($EGFR_{IHC}$) obtained using immunohistochemistry (IHC). Correlation between results obtained by the two different techniques was highly significant ($r_s = 0.63$, $P < 0.001$, $n = 190$). This correlation improved even further ($r_s = 0.76$) when sections were estimated using an IHC score that took into account percentage staining, intensity and relative tumour area. Furthermore, the relationship between clinicopathological features and prognosis was identical for the two methods. The expression of EGFR was highest in squamous cell carcinomas, but it was not correlated with other characteristics such as age, sex, histological grading, stage or prognosis. We conclude that evaluation of EGFR content using IHC and ELISA produces comparable results.

**Keywords:** epidermal growth factor receptor; enzyme-linked immunosorbent assay; immunohistochemistry, non-small-cell lung cancer; prognosis

Epidermal growth factor receptor (EGFR) is the protein product of the proto-oncogene *HER-1*. Ligand binding to the extracellular region of EGFR causes receptor dimerization, resulting in autophosphorylation and activation of cytoplasmic signal protein, which ultimately triggers DNA synthesis associated with proliferation and differentiation (Prigent and Lemoine, 1992). However, whether aberrant expression of EGFR is causally or consequently related to the development of cancer remains to be established. Some studies have reported EGFR to be of prognostic importance in lung cancer (Volm and Mattern, 1993, Volm et al, 1993); unfortunately, the relationship to survival has been inconsistent (Pfeiffer et al, 1996a).

Immunohistochemistry (IHC) is the most widely applied technique to assess expression of EGFR in patients with non-small-cell lung cancer (NSCLC) (Pfeiffer et al, 1996*a*). Immunohistochemical results depend on the primary antibody, the visualization system and whether fixed or frozen tissues are used. Furthermore, the evaluation of immunohistochemical slides is inherently qualitative or, at best, semiquantitative. The enzyme-linked immunosorbent assay (ELISA) can quantify EGFR in cytosol extracts. We used a recently developed ELISA technique (Christensen et al, 1995) to measure the level of EGFR in a large cohort of lung cancer patients and compared the results with immunohistochemical analysis on cryosections and with clinical and pathological data. To our knowledge, this is the first study to compare the detection of EGFR using IHC and ELISA.

*Received 30 April 1997*
*Revised 20 January 1998*
*Accepted 30 January 1998*

*Correspondence to:* P Pfeiffer, Department of Oncology R, Odense University Hospital, DK-5000 Odense C, Denmark

### MATERIAL AND METHODS

### Patients and tumour samples

Frozen tumour tissue from 190 previously untreated lung cancer patients (median age 61 years (range 42–79 years); 131 men and 59 women) were available for ELISA and IHC. All the patients were surgically treated at Odense University Hospital, Denmark, between 1984 and 1991; median follow-up was 66 months (range 39–119 months). The study was approved by the local ethics committee.

We analysed the relationship between EGFR and histological subtype and the correlation between IHC and ELISA for all lung cancer patients; however, all other analyses were restricted to 180 patients with NSCLC. The TNM stage of NSCLC was determined retrospectively, according to the new International Staging System for lung cancer (Mountain, 1987). No patient received post-operative adjuvant cytotoxic or radiation therapy.

### Tissue preparation

Lung resections were received unfixed in the pathology laboratory, immediately after surgical removal. One piece of tumour measuring approximately 1 $cm^3$ was cut out and divided in two. One part was placed in cryoconservation tubes, snap-frozen at –80°C, and stored until further examinations. The other part was formalin fixed and embedded in paraffin for histological classification.

**Figure 1** (**A**) Measurement of EGFR content using ELISA ($EGFR_{ELISA}$) in 190 patients with lung cancer. (**B**) Estimation of EGFR content using IHC ($EGFR_{IHC}$) in 190 patients with lung cancer

### ELISA

Biopsies were stored at –80°C until further analysis. We used a recently developed ELISA technique to measure EGFR content ($EGFR_{ELISA}$) (Christensen et al, 1995). Briefly, biopsies (median 44 mg; range 5–192 mg) from 190 patients were cut, ultrasonicallyhomogenized in buffer and centrifuged at 20 000 *g*. The membrane pellet was resuspended in buffer and the receptor was solubilized by incubation with 2% Triton X-100, followed by centrifugation at 20 000 *g*. EGFR was measured in the supernatant. We used EGFR1 (RPN 513; Amersham, UK) as catching antibody and biotinylated Ab-1 (GR01; Oncogene Science, USA) as detecting antibody. EGFR from placenta membranes was used as the calibration standard. The detection limit was 0.08 nmol $l^{-1}$.

### Immunohistochemical analysis

EGFR immunostaining was performed on 5-µm cryostat sections, using the peroxidase labelled Streptavidin–Biotin (LSAB) technique and EGFR1 (Amersham, UK) as primary antibody (Pfeiffer et al, 1996*a*, *b*).

**Figure 2** (**A**) Correlation between $EGFR_{IHC}$ and $EGFR_{ELISA}$ in 190 patients with lung cancer ($r_s$ = 0.63, $P < 0.001$). (**B**) Correlation between an IHC score (immunohistochemical score combining intensity, number of stained tumour cells and relative tumour area) and $EGFR_{ELISA}$ ($r_s$ = 0.76; $P < 0.0001$).
(**A**) $r_s$ = 0.63; (**B**) $r_s$ = 0.76

### Immunohistochemical assessment

The percentage of positively reacting tumour cells ($EGFR_{IHC}$: 0–100%) was estimated by one author (PP) (Pfeiffer et al, 1996*a*).

An immunohistochemical score (IHC score), which took into account percentage staining, intensity and relative tumour area, was also calculated. The average intensity of staining ($INT_{SCO}$) was given a score between 0 and 3. The number of positively stained tumour cells ($CELL_{SCO}$) was also scored between 0 and 3: 0; 1 (1–49%); 2 (50–79%); 3 (80–100%). In addition, the relative tumour area was assessed by evaluating the proportion of malignant cells compared with the entire histological section ($AREA_{SCO}$) and expressed as a proportion (0–1.0). The IHC score was then

© Cancer Research Campaign 1998

**Table 1** Relationship between $EGFR_{ELISA}$ content and histological subtype in 190 patients with lung cancer.

| | | $EGFR_{ELISA}$ content | | |
|---|---|---|---|---|
| | *n* | Low (%) | Medium (%) | High (%) |
| SqC | 100 | 37 | 27 | 36 |
| AdC | 57 | 74 | 10 | 16 |
| LaC | 23 | 61 | 22 | 17 |
| Carcinoid | 9 | 100 | 0 | 0 |
| SCLC | 1 | 100 | 0 | 0 |
| Total | 190 | 54 | 20 | 26 |

NSCLC > SCLC + carcinoid, $P < 0.001$; SqC > AdC, $P < 0.00001$; SqC > LaC, $P = 0.01$. $EGFR_{ELISA}$, EGFR content measured using ELISA; SqC, squamous cell carcinoma; AdC, adenocarcinoma; LaC, large cell carcinoma; T, primary tumour; N, lymph node.

**Figure 3** Survival of 180 patients with NSCLC according to measurement of EGFR using ELISA (low, medium or high) ($P = 0.6$). ...., low $n = 89$; - - -, medium $n = 42$; —, high $n = 49$

assigned to each tumour by multiplying $INT_{SCO}$, $CELL_{SCO}$ and $AREA_{SCO}$; the IHC score thus ranged from 0 to 9.0.

### Statistical evaluation

All *P*-values are from two-sided tests. Statistical significance was claimed for *P*-values less than or equal to 0.05. Relative risk (RR) is followed by 95% confidence intervals (CI). The association between two variables was quantified using Spearman's rank correlation coefficient ($r_s$) test.

Survival curves were estimated according to the Kaplan–Meier method (Kaplan and Meier, 1958) and compared using the log-rank test (Peto et al, 1977) or, if there were three or more ordered categories, the log-rank test for trend. BMDP/PC Release 7.01 (BMDP Statistical Software, 1993, Los Angeles, CA, USA) was used for all statistical analyses.

### RESULTS

The median $EGFR_{ELISA}$ was 1.0 nmol EGFR $g^{-1}$ protein (range 0.1–26.9 nmol EGFR $g^{-1}$ protein). This value was selected as a cut-off point and a second cut-off point (2.0 nmol EGFR $g^{-1}$ protein) was arbitrarily chosen (Figure 1A). The patients were thus categorized as low (< 1.0 nmol EGFR $g^{-1}$ protein), medium (1.0–2.0 nmol EGFR $g^{-1}$ protein) or high (> 2.0 nmol EGFR $g^{-1}$ protein).

The distribution of $EGFR_{IHC}$ is shown in Figure 1B; the median $EGFR_{IHC}$ was 80%. $EGFR_{ELISA}$ and $EGFR_{IHC}$ results of paired samples from 190 patients with lung cancer are shown in Figure 2A. There was a highly significant correlation between $EGFR_{IHC}$ and $EGFR_{ELISA}$ ($r_s = 0.63$, $P < 0.001$). The association between ELISA and IHC was improved even further ($r_s = 0.76$) when the IHC score was used, i.e. when we also took into account the intensity of staining and relative tumour area (Figure 2B).

The association between EGFR expression and other variables was similar whether we used results obtained using ELISA or IHC. We found expression of EGFR in all subtypes of NSCLC, but most frequently in squamous cell carcinomas (Table 1).

$EGFR_{ELISA}$ or $EGFR_{IHC}$ was not correlated with age, tumour size, T status, lymph node involvement, stage, histological grading or time of diagnosis. Furthermore, squamous cell carcinoma was the only variable that correlated with EGFR expression in a logistic regression model. The relative risk for high $EGFR_{ELISA}$ and $EGFR_{IHC}$ content for squamous cell carcinoma was 4.1 (95% CI 2.2–7.7) and 4.4 (95% CI 2.3–8.3), respectively.

We did not find any correlation between EGFR expression and survival in the entire group of patients with NSCLC (Figure 3), nor in any subgroup analysis.

### DISCUSSION

Most studies on NSCLC have focussed on the protein level. Berger et al (1987) found a close correlation between IHC and autophosphorylation activity. Ligand-binding studies have shown that EGFR-binding characteristics were comparable in tumour and normal lung tissue (Hwang et al, 1986; Veale et al, 1989; Dittadi et al, 1991). These studies point to the fact that, if expressed in NSCLC, a normal functional EGFR is operating. Thus, this receptor can be detected by antibodies that recognize EGFR, using either ELISA or IHC.

It is often stated that patients with overexpression of EGFR have a shorter survival, but in a prior study (Pfeiffer et al, 1996*a*) we were unable to find a correlation between EGFR and prognosis. To substantiate these findings, we measured EGFR content in frozen tumour samples using a recently developed ELISA technique (Christensen et al, 1995). We found a highly significant correlation between $EGFR_{IHC}$ and $EGFR_{ELISA}$, and also an identical correlation with other variables. A high $EGFR_{ELISA}$ value always corresponded with a high $EGFR_{IHC}$ value, whereas samples with a high $EGFR_{IHC}$ showed variable values for $EGFR_{ELISA}$. To interpret this discrepancy, one must realize that even although $EGFR_{ELISA}$ is a quantitative measure, ELISA calculates an average EGFR content in a homogenized tissue sample consisting of tumour tissue intermixed with various amounts of non-tumour tissue, including normal lung tissue, connective tissue and necrotic tissue. Another inconvenience is that it requires handling of fresh or frozen tissue. The major advantages of ELISA are its quantitative nature and the use of a calibration curve (Christensen et al, 1995).

Quantitation of EGFR has been reported in a few prior studies. Veale et al (1993) measured EGFR using the ligand-binding assay in 19 selected patients with NSCLC. They found that EGFR quantitation may give prognostic information and proposed confirmation in a larger prospective study. Recent studies have shown that

© Cancer Research Campaign 1998

EGFR content can be quantitated on tumour sections (Stanton et al, 1994; Robertson et al, 1996), but whether quantitative analysis will add to the prognostic significance of EGFR is doubtful. Furthermore, it is still complex to interpret results in heterogeneous tumours, i.e. what is the biological importance of a large amount of EGFR in few tumour cells compared with a lower amount in most tumour cells.

IHC represents a small sectional view of a larger tissue area; the result relies very much on the immunohistochemical technique, and the results are subjective and qualitative. The major advantage of the immunohistochemical technique is the maintenance of tissue architecture and in situ localization of the antigen. By contrast with ELISA, in which cancer cells cannot be distinguished from non-malignant tissue, this distinction can easily be made using immunohistochemical analysis. Also, IHC is able to identify tumour positivity, even in very small tumour samples.

When we took into account the percentage of stained tumour cells, intensity of staining and cellularity, the correlation between ELISA and IHC was further improved. At least some of the remaining discrepancy may be due to intratumoral heterogeneity. To our knowledge, a detailed comparison of EGFR expression using ELISA and IHC has not been published previously, but some studies have determined the related growth factor receptor $p185^{HER-2}$ using IHC and ELISA (Dawkins et al, 1993; Dittadi et al, 1993; Nugent et al, 1994; Piffanelli et al, 1996). In brief, the overall agreement was comparable with that of the present study.

In agreement with most other studies, we found expression of EGFR ($EGFR_{ELISA}$ or $EGFR_{IHC}$) in all subtypes of NSCLC, but most frequently in squamous cell carcinomas, and no correlation between EGFR expression and the size of the primary tumour, lymph node status or stage. The expression of EGFR without correlation to stage suggests an important step during early tumour genesis. It might provide the potential tumour cell with the ability continually to proliferate when the supply of growth factors is restricted and/or escape terminal differentiation.

In conclusion, detection of EGFR using IHC and ELISA produces comparable results, particularly when IHC is estimated using an immunohistochemical score that evaluates percentage staining, intensity and relative tumour area, however further methodological standardization is needed. Expression of EGFR was found in all histological subtypes of NSCLC, but especially in squamous cell carcinoma. Quantitative or qualitative EGFR expression was not correlated with extension of tumour tissue or histological grading and was without prognostic value in patients with NSCLC.

### ACKNOWLEDGEMENT

This study was supported by grants from the Boel Foundation, the Danish Medical Research Council, and the Danish Cancer Society. We thank Lisbeth Jensen and May-Britt Berg for their technical assistance.

### REFERENCES

Berger MS, Gullick WJ, Greenfield C, Evans S, Addis BJ and Waterfield MD (1987) Epidermal growth factor receptors in lung tumours. *J Pathol* **152**: 297–307

Christensen ME, Engbaek F, Therkildsen MH, Bretlau P and Nexo E (1995) A sensitive enzyme-linked immunosorbent assay used for quantitation of epidermal growth factor receptor protein in head and neck carcinomas: evaluation, interpretations and limitations. *Br J Cancer* **71**: 1487–1493

Dawkins HJ, Robbins PD, Sarna M, Carrello S, Harvey JM and Sterrett GF (1993) c-erbB-2 amplification and overexpression in breast cancer: evaluation and comparison of Southern blot, slot blot, ELISA and immunohistochemistry. *Pathology* **25**: 124–132

Dittadi R, Gion M, Pagan V, Brazzale A, Del Maschio O, Bargossi A, Busetto A and Bruscagnin G (1991) Epidermal growth factor receptor in lung malignancies. Comparison between cancer and normal tissue. *Br J Cancer* **64**: 741–744

Dittadi R, Catozzi L, Gion M, Brazzale A, Capitanio G, Gelli MC, Menegon A, Gardini G, Malagutti R and Piffanelli A (1993) Comparison between western blotting, immunohistochemical and ELISA assay for p185neu quantitation in breast cancer specimens. *Anticancer Res* **13**: 1821–1824

Hwang DL, Tay YC, Lin SS and Lev Ran A (1986) Expression of epidermal growth factor receptors in human lung tumors. *Cancer* **58**: 2260–2263

Kaplan EL and Meier P (1958) Nonparametric estimation from incomplete observations. *J Am Statist Assoc* **53**: 457–481

Mountain CF (1987) The new international staging system for lung cancer. *Surg Clin N Am* **67**: 925–935

Nugent A, Gallagher J, Dolan J, O'Higgins N and Duffy MJ (1994) Assay of the c-erbB-2 oncogene encoded protein by ELISA and immunocytochemistry in human breast cancer. *Ann Clin Biochem* **31**: 171–173

Peto R, Pike MC, Armitage P, Breslow NE, Cox DR, Howard SV, Mantel N, McPherson K, Peto J and Smith PG (1977) Design and analysis of randomized clinical trials requiring prolonged observation of each patient. II. Analysis and examples. *Br J Cancer* **35**: 1–39

Pfeiffer P, Clausen PP, Andersen K and Rose C (1996*a*) Lack of prognostic significance of epidermal growth factor receptor and the oncoprotein p185HER-2 in patients with systemically untreated non-small-cell lung cancer: an immunohistochemical study on cryosections. *Br J Cancer* **74**: 86–91

Pfeiffer P, Grabau DA, Nielsen O and Clausen PP (1996*b*) Immunohistochemical bulk staining of slides using a rack peroxidase-labelled streptavidin-biotin technique. *Appl Immunohistochem* **4**: 135–138

Piffanelli A, Dittadi R, Catozzi L, Gion M, Capitanio G, Gelli MC, Brazzale A, Malagutti R, Pelizzola D, Menegon A, Giovannini G and Gardini G (1996) Determination of ErbB2 protein in breast cancer tissues by different methods. Relationship with other biological parameters. *Breast Cancer Res Treat* **37**: 267–276

Prigent SA and Lemoine NR (1992) The type 1 (EGFR-related) family of growth factor receptors and their ligands. *Prog Growth Factor Res* **4**: 1–24

Robertson KW, Reeves JR, Smith G, Keith WN, Ozanne BW, Cooke TG and Stanton PD (1996) Quantitative estimation of epidermal growth factor receptor and c-erbB-2 in human breast cancer. *Cancer Res* **56**: 3823–3830

Stanton P, Richards S, Reeves J, Nikolic M, Edington K, Clark L, Robertson G, Souter D, Mitchell R, Hendler FJ, Cooke T, Parkinson EK and Ozanne BW (1994) Epidermal growth factor receptor expression by human squamous cell carcinomas of the head and neck, cell lines and xenografts. *Br J Cancer* **70**: 427–433

Veale D, Kerr N, Gibson GJ and Harris AL (1989) Characterization of epidermal growth factor receptor in primary human non-small cell lung cancer. *Cancer Res* **49**: 1313–1317

Veale D, Kerr N, Gibson GJ, Kelly PJ and Harris AL (1993) The relationship of quantitative epidermal growth factor receptor in non-small-cell lung cancer to long term survival. *Br J Cancer* **68**: 162–165

Volm M and Mattern J (1993) Correlation between successful heterotransplantation of lung tumors in nude mice, poor prognosis of patients and expression of Fos, Jun, ErbB1, and Ras. *Anticancer Res* **13**: 2021–2025

Volm M, Drings P and Wodrich W (1993) Prognostic significance of the expression of c-fos, c-jun and c-erbB-1 oncogene products in human squamous cell lung carcinomas. *J Cancer Res Clin Oncol* **119**: 507–510


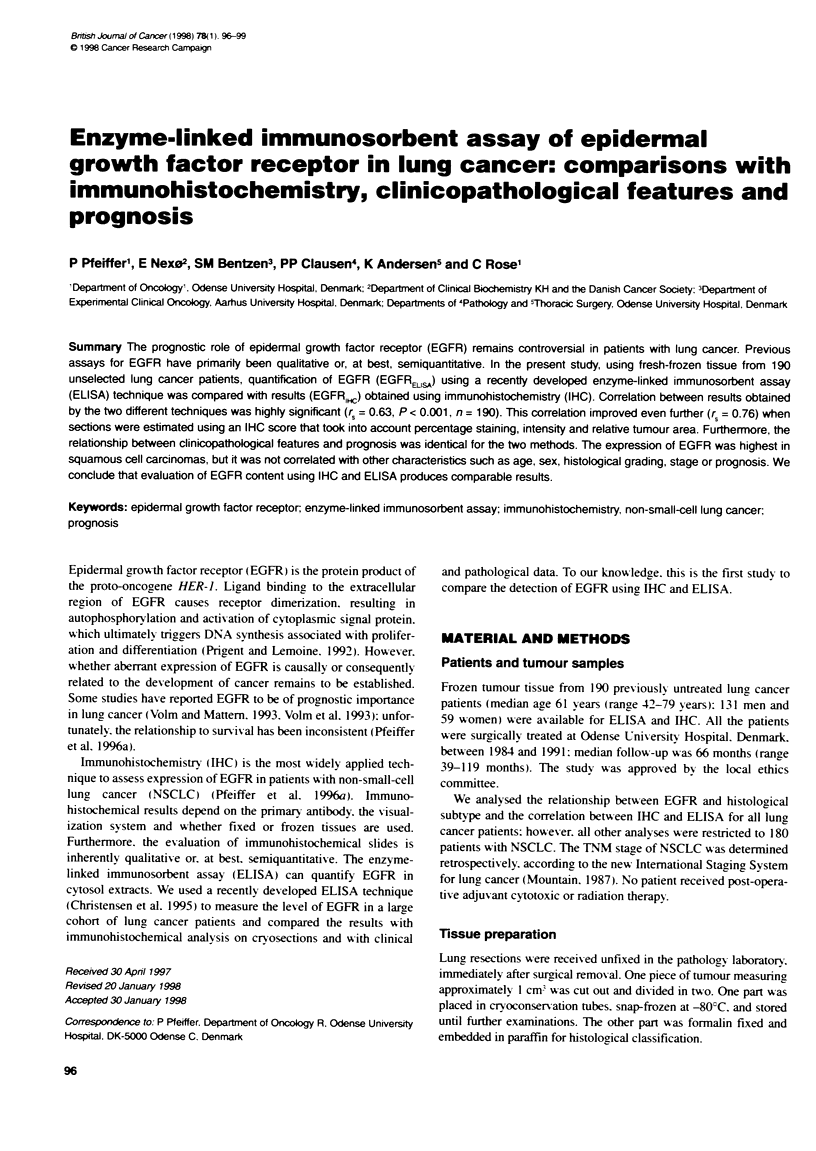

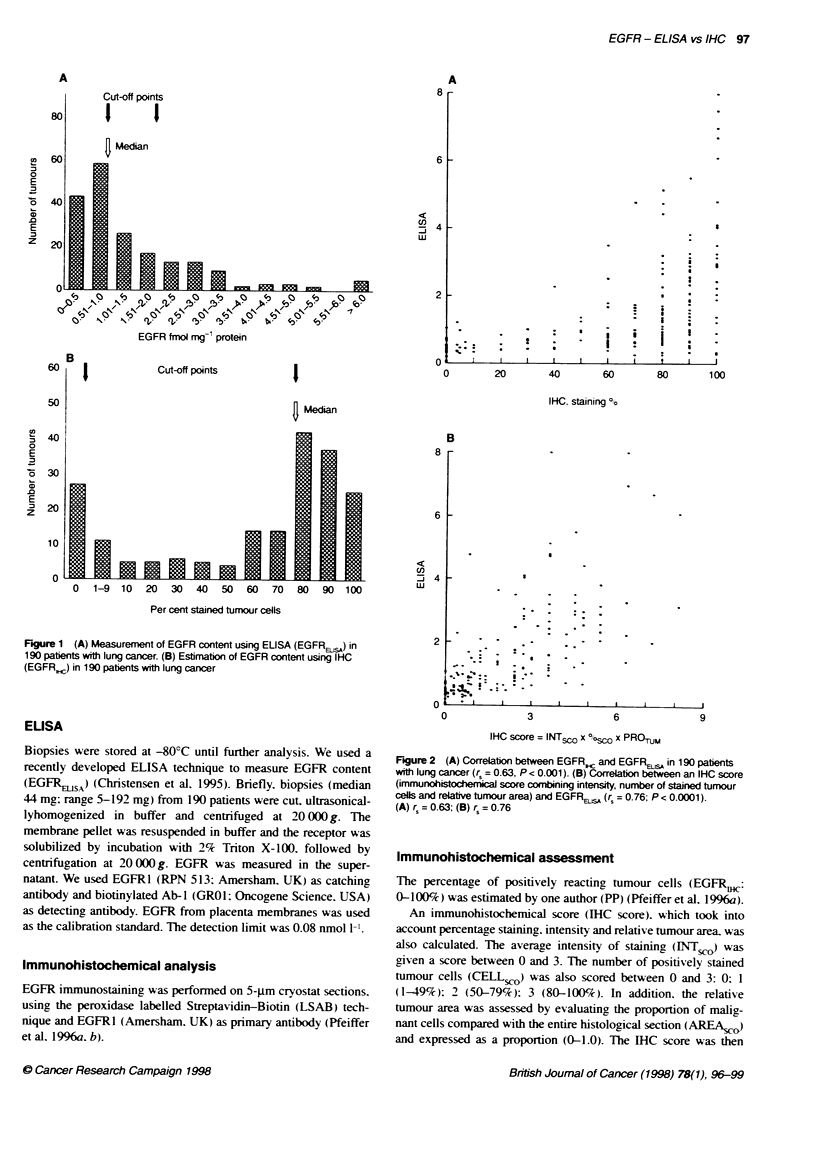

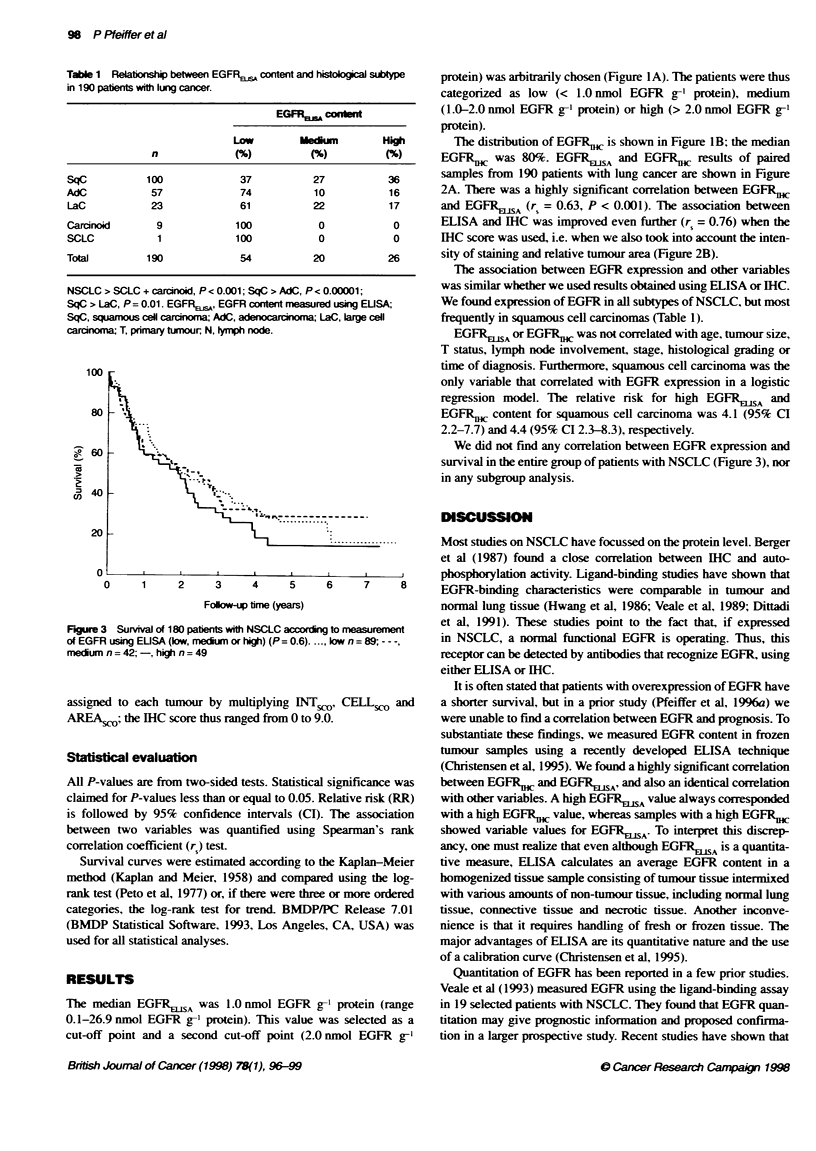

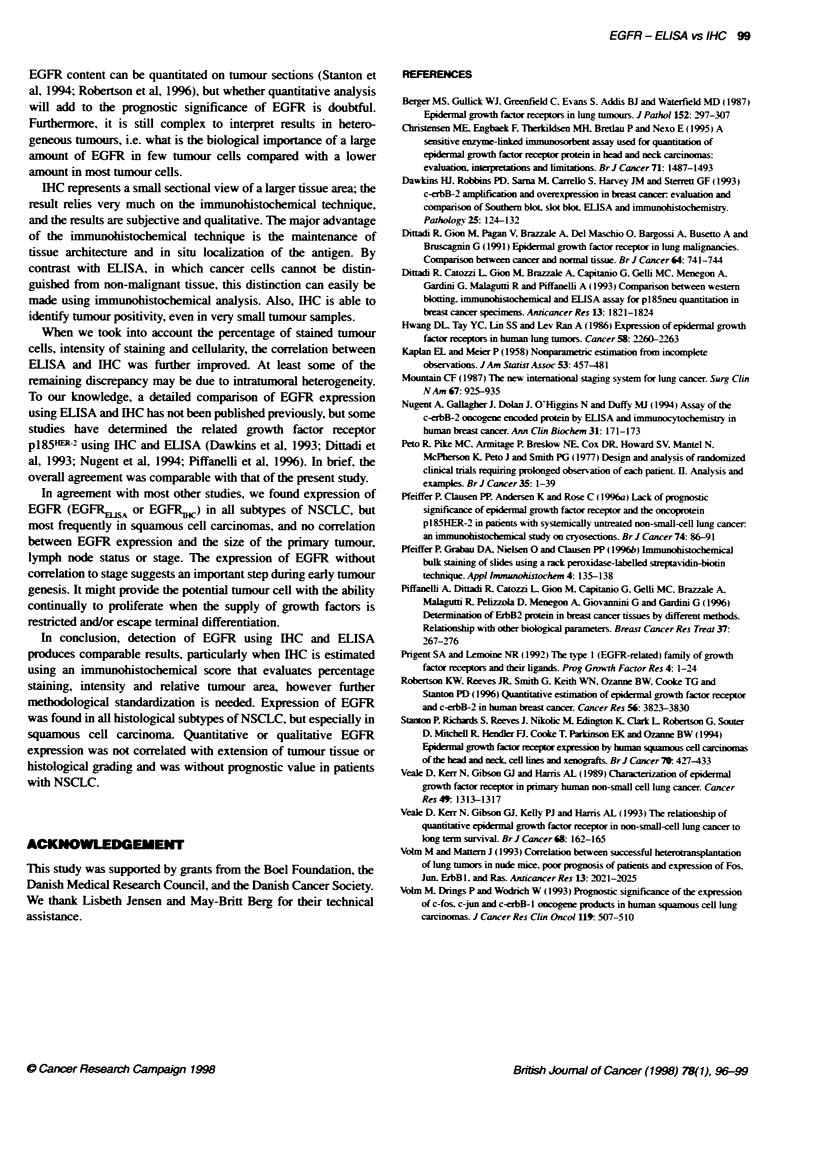


## References

[OCR_00467] Berger M. S., Gullick W. J., Greenfield C., Evans S., Addis B. J., Waterfield M. D. (1987). Epidermal growth factor receptors in lung tumours.. J Pathol.

[OCR_00468] Christensen M. E., Engbaek F., Therkildsen M. H., Bretlau P., Nexø E. (1995). A sensitive enzyme-linked immunosorbent assay used for quantitation of epidermal growth factor receptor protein in head and neck carcinomas: evaluation, interpretations and limitations.. Br J Cancer.

[OCR_00474] Dawkins H. J., Robbins P. D., Sarna M., Carrello S., Harvey J. M., Sterrett G. F. (1993). c-erbB-2 amplification and overexpression in breast cancer: evaluation and comparison of Southern blot, slot blot, ELISA and immunohistochemistry.. Pathology.

[OCR_00484] Dittadi R., Catozzi L., Gion M., Brazzale A., Capitanio G., Gelli M. C., Menegon A., Gardini G., Malagutti R., Piffanelli A. (1993). Comparison between western blotting, immunohistochemical and ELISA assay for p185neu quantitation in breast cancer specimens.. Anticancer Res.

[OCR_00482] Dittadi R., Gion M., Pagan V., Brazzale A., Del Maschio O., Bargossi A., Busetto A., Bruscagnin G. (1991). Epidermal growth factor receptor in lung malignancies. Comparison between cancer and normal tissue.. Br J Cancer.

[OCR_00490] Hwang D. L., Tay Y. C., Lin S. S., Lev-Ran A. (1986). Expression of epidermal growth factor receptors in human lung tumors.. Cancer.

[OCR_00498] Mountain C. F. (1987). The new International Staging System for Lung Cancer.. Surg Clin North Am.

[OCR_00504] Nugent A., Gallagher J., Dolan J., O'Higgins N., Duffy M. J. (1994). Assay of the c-erbB-2 oncogene encoded protein by ELISA and immunocytochemistry in human breast cancer.. Ann Clin Biochem.

[OCR_00509] Peto R., Pike M. C., Armitage P., Breslow N. E., Cox D. R., Howard S. V., Mantel N., McPherson K., Peto J., Smith P. G. (1977). Design and analysis of randomized clinical trials requiring prolonged observation of each patient. II. analysis and examples.. Br J Cancer.

[OCR_00513] Pfeiffer P., Clausen P. P., Andersen K., Rose C. (1996). Lack of prognostic significance of epidermal growth factor receptor and the oncoprotein p185HER-2 in patients with systemically untreated non-small-cell lung cancer: an immunohistochemical study on cryosections.. Br J Cancer.

[OCR_00525] Piffanelli A., Dittadi R., Catozzi L., Gion M., Capitanio G., Gelli M. C., Brazzale A., Malagutti R., Pelizzola D., Menegon A. (1996). Determination of ErbB2 protein in breast cancer tissues by different methods. Relationships with other biological parameters.. Breast Cancer Res Treat.

[OCR_00533] Prigent S. A., Lemoine N. R. (1992). The type 1 (EGFR-related) family of growth factor receptors and their ligands.. Prog Growth Factor Res.

[OCR_00539] Robertson K. W., Reeves J. R., Smith G., Keith W. N., Ozanne B. W., Cooke T. G., Stanton P. D. (1996). Quantitative estimation of epidermal growth factor receptor and c-erbB-2 in human breast cancer.. Cancer Res.

[OCR_00544] Stanton P., Richards S., Reeves J., Nikolic M., Edington K., Clark L., Robertson G., Souter D., Mitchell R., Hendler F. J. (1994). Epidermal growth factor receptor expression by human squamous cell carcinomas of the head and neck, cell lines and xenografts.. Br J Cancer.

[OCR_00549] Veale D., Kerr N., Gibson G. J., Harris A. L. (1989). Characterization of epidermal growth factor receptor in primary human non-small cell lung cancer.. Cancer Res.

[OCR_00554] Veale D., Kerr N., Gibson G. J., Kelly P. J., Harris A. L. (1993). The relationship of quantitative epidermal growth factor receptor expression in non-small cell lung cancer to long term survival.. Br J Cancer.

[OCR_00564] Volm M., Drings P., Wodrich W. (1993). Prognostic significance of the expression of c-fos, c-jun and c-erbB-1 oncogene products in human squamous cell lung carcinomas.. J Cancer Res Clin Oncol.

[OCR_00559] Volm M., Mattern J. (1993). Correlation between successful heterotransplantation of lung tumors in nude mice, poor prognosis of patients and expression of Fos, Jun, ErbB1, and Ras.. Anticancer Res.

